# Pretarsal Augmentation With PLLA‐b‐PEG/HA Filler: Anatomical Plane and Safety Considerations

**DOI:** 10.1111/jocd.71099

**Published:** 2026-07-22

**Authors:** Chuan‐Yuan Lin, Jui‐Yu Lin

**Affiliations:** ^1^ Nanzi Annyeong Clinic Kaohsiung City Taiwan; ^2^ Chengdu Royalty Aesthetic Clinic Chengdu Sichuan China; ^3^ Hangzhou Royalty Aesthetic Clinic Hangzhou Zhejiang China

**Keywords:** aegyo‐sal, injection plane, periorbital rejuvenation, PLLA‐b‐PEG, poly‐L‐lactic acid, pretarsal augmentation

To the Editor,

1

We read with great interest the article by Chen et al. [1], which evaluated a cross‐linked sodium hyaluronate filler containing poly‐L‐lactic acid‐b‐poly(ethylene glycol) (PLLA‐b‐PEG) microspheres for periorbital rejuvenation. The authors should be commended for addressing this technically demanding region through physicochemical characterization, preclinical histology, and clinical follow‐up. One point deserves further anatomical discussion. The authors correctly describe pretarsal fullness, or “aegyo‐sal,” as an aesthetic feature valued in East Asian patients and as a bulge of the orbicularis oculi muscle (OOM) [1]. In their standardized protocol, pretarsal fullness was treated in the “subdermal” layer using a 27G cannula with linear threading and a unilateral dose of 0.3–1.0 mL [1]. However, the anatomical meaning and safety implications of “subdermal” placement in the pretarsal lower eyelid require clarification.

The pretarsal lower eyelid is anatomically distinct from facial regions with a more developed subcutaneous compartment. Eyelid skin is extremely thin, and in the pretarsal region the dermis, pretarsal OOM, and tarsal plate are closely apposed (Figure 1) [2, 3]. Therefore, a cannula advanced near the lower lash line may not reliably remain in a true plane immediately beneath the dermis and superficial to the OOM. The actual deposition may instead be partially intramuscular or located in a sub‐orbicularis/supratarsal plane. These planes are not anatomically equivalent. This distinction is clinically relevant because the material contains PLLA‐b‐PEG microspheres suspended in hyaluronic acid (HA) gel and is intended to provide both immediate filling and delayed tissue stimulation [1]. Prior experience with conventional PLLA cannot be directly extrapolated to this PEG‐modified PLLA/HA formulation. Nevertheless, PLLA literature has suggested that placement in or through active muscles, particularly under the eye or near the mouth, may contribute to localized overcorrection or nodularity, possibly reflecting product trapped among muscle fibers [4, 5]. Periorbital PLLA‐related granulomas have also been reported [6]. These observations should be interpreted only as cautionary background, not as direct evidence against the present product.

**FIGURE 1:**
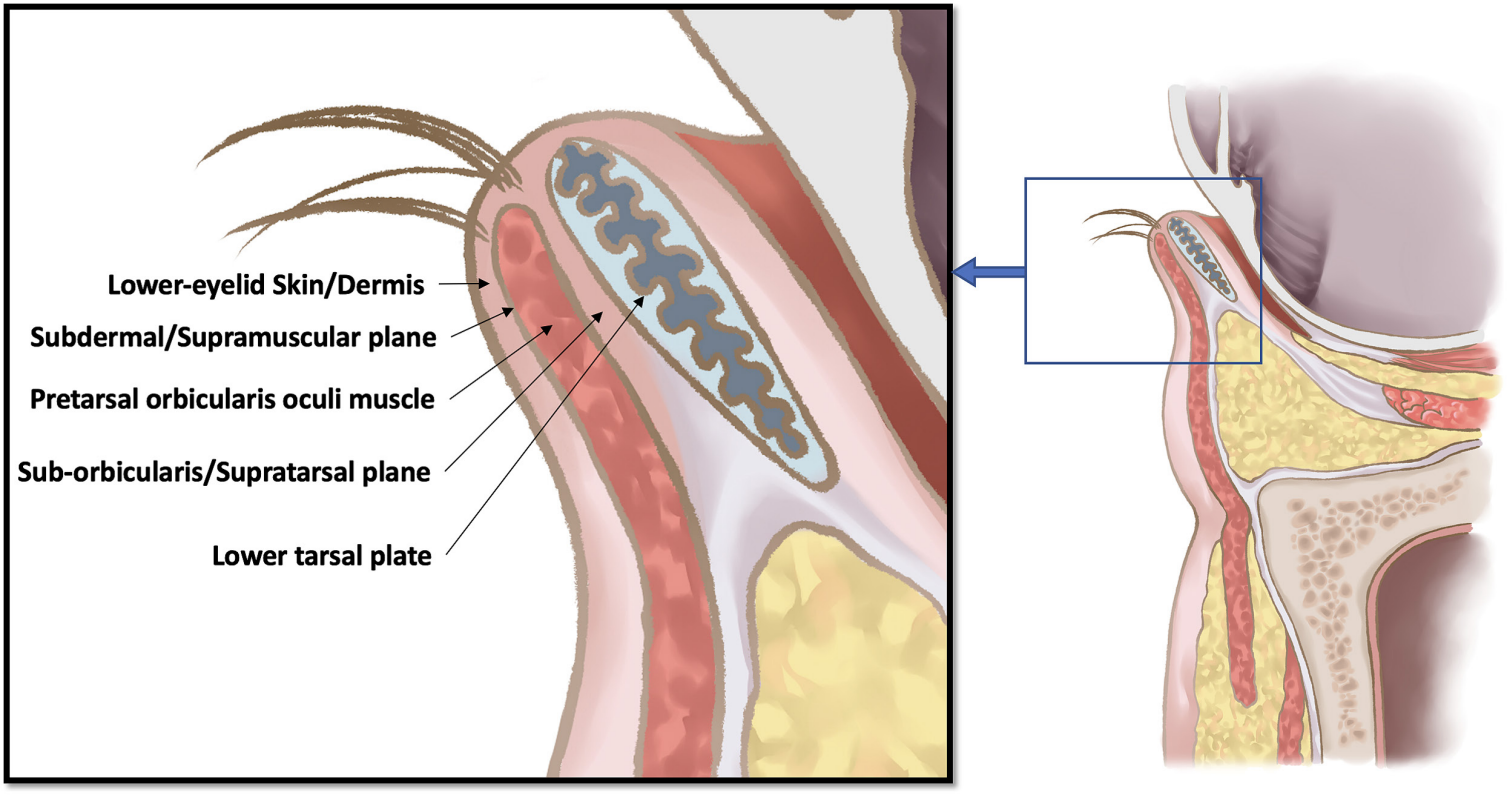
Schematic sagittal anatomy of the pretarsal lower eyelid. The lower‐eyelid skin/dermis, pretarsal orbicularis oculi muscle, and lower tarsal plate are closely apposed, with minimal intervening subcutaneous tissue. A cannula described as passing in a “subdermal” plane may therefore result in true subdermal/supramuscular, partially intramuscular, or sub‐orbicularis/supratarsal deposition.

The absence of nodules or progressive contour irregularities in the present series is reassuring, but several interpretations are possible. First, the treating injector may have used a refined technique to maintain the product in a truly subdermal or supramuscular plane despite the limited pretarsal tissue thickness. Second, partial intramuscular deposition may have occurred, but the PEG‐modified PLLA/HA formulation may have lower nodulogenic potential than conventional PLLA. Third, partial intramuscular deposition may have occurred, but the small cohort size, limited number of pretarsal cases, or limited exposure volume may have been insufficient to reveal rare or delayed nodular events. These possibilities cannot be distinguished without objective confirmation of the actual deposition plane.

Future studies should clarify not only the anatomical plane achieved during pretarsal augmentation but also the explanation for the favorable safety findings observed in the present series. High‐frequency ultrasound, cadaveric injection studies, or immediate post‐injection imaging may help determine whether the filler remains superficial to the OOM or is deposited within an intramuscular or sub‐orbicularis/supratarsal plane. If true subdermal or supramuscular placement is achievable, future reports should describe the technical details that allow reliable maintenance of this narrow plane. If partial intramuscular deposition occurs, further investigation should determine whether the PEG‐modified PLLA/HA formulation has a lower tendency toward nodularity than conventional PLLA when placed in or near active muscle. Finally, larger prospective cohorts with standardized reporting of pretarsal injection volume, number of treated pretarsal cases, dynamic lower‐eyelid assessment, and longer follow‐up are needed to determine whether rare or delayed contour irregularities may emerge with greater clinical exposure.

## Funding

The authors have nothing to report.

## Ethics Statement

The authors have nothing to report.

## Conflicts of Interest

The authors declare no conflicts of interest.

## Data Availability

Data sharing is not applicable to this article because no datasets were generated or analyzed.
